# Capturing SARS-CoV-2 from patient samples with low viral abundance: a comparative analysis

**DOI:** 10.1038/s41598-022-23422-3

**Published:** 2022-11-11

**Authors:** Juliana Pipoli da Fonseca, Etienne Kornobis, Elodie Turc, Vincent Enouf, Laure Lemée, Thomas Cokelaer, Marc Monot

**Affiliations:** 1 Institut Pasteur, Université Paris Cité, Plate-Forme Technologique Biomics, 75015 Paris, France; 2 National Reference Center for Respiratory Viruses, Molecular Genetics of RNA Viruses, CNRS-UMR 3569, The Institut Pasteur, Paris, France; 3 Mutualized Platform of Microbiology, Pasteur International Bioresources Network, The Institut Pasteur, Paris, France; 4 Institut Pasteur, Université Paris Cité, Bioinformatics and Biostatistics Hub, 75015 Paris, France

**Keywords:** Biological techniques, Molecular biology, Diseases

## Abstract

Since the beginning of the SARS-CoV-2 coronavirus pandemic, genome sequencing is essential to monitor viral mutations over time and by territory. This need for complete genetic information is further reinforced by the rapid spread of variants of concern. In this paper, we assess the ability of the hybridization technique, Capture-Seq, to detect the SARS-CoV-2 genome, either partially or in its integrity on patients samples. We studied 20 patient nasal swab samples broken down into five series of four samples of equivalent viral load from CT25 to CT36+ . For this, we tested 3 multi-virus panel as well as 2 SARS-CoV-2 only panels. The panels were chosen based on their specificity, global or specific, as well as their technological difference in the composition of the probes: ssRNA, ssDNA and dsDNA. The multi-virus panels are able to capture high-abundance targets but fail to capture the lowest-abundance targets, with a high percentage of off-target reads corresponding to the abundance of the host sequences. Both SARS-CoV-2-only panels were very effective, with high percentage of reads corresponding to the target. Overall, capture followed by sequencing is very effective for the study of SARS-CoV-2 in low-abundance patient samples and is suitable for samples with CT values up to 35.

## Introduction

The agent of Covid-19, the SARS-CoV-2 coronavirus, is the etiological cause of a severe acute respiratory syndrome. In 2020, mortality rates steadily increased worldwide, resulting in the designation of SARS-CoV-2 as a global challenge to health and the economy. During the same year, vaccines were developed in response to this pandemic, including those based on new technologies, such as the mRNA Pfizer and Moderna vaccines^1^. However, testing strategies are still essential to public health policy responses to the Covid-19 pandemic. There are essentially two main technologies available to detect SARS-CoV-2 in genetic samples: those based on molecular tests or those based on rapid antigen tests. Quantitative real-time reverse‐transcriptase polymerase chain reaction (RT‐qPCR) assays are recommended for the standard diagnosis of SARS‐CoV‐2 infection^2^. Following this initial test, genome sequencing is essential to monitor viral mutations over time and by territory^3^. This need for complete genetic information is further reinforced by the rapid spread of variants of concern, such as B1.1.7^4^, B1.617.2^5^, and B1.1.529^6^. Arguably, the least biased approach to obtain SARS-CoV-2 genomes is to carry out direct RNA sequencing. This could be potentially achieved using the Oxford Nanopore Technologies platform^7^. There are, however, important limitations, such as the large amount of starting material required and the high error rate, which could require additional sequencing. Depending on the viral load detected, metagenomic sequencing or amplicon methods are most often used.

The National Reference Centers (NRCs) for Respiratory Viruses in France (Institut Pasteur, Paris, and Hospices Civils de Lyon, France) have set up a common strategy: up to a pre-defined RT-qPCR cycle threshold (CT) of 25, standard metagenomic sequencing is carried out (with a yield of 20M reads per sample); above this threshold, sequencing is based on amplicons^8^. During the pandemic, we propose to set up a third and complementary approach using hybridization-capture sequencing. In spite of its higher cost and technical complexity, this approach provides several advantages. First, adding capture sequencing to existing NGS platform protocols is straightforward. Second, the low sequencing depth required (1M reads per sample) reduces the sequencing costs and simplifies both data management and turnaround time. Other advantages are its robustness (hybridization capture tolerates both rearrangements and sequence variation) and adaptability (the addition of probes is possible to follow the evolution of the virus).

The very high sensitivity of hybridization can allow the detection of the virus genome in samples considered to be negative by RT-qPCR (CT > 36 for NRCs) for patients who present typical clinical symptoms^9^. Diverse sampling also does not appear to affect the results, as shown in the studies published to date using the capture-sequencing method on nasopharyngeal^10,11^, throat^10,12^, and anal^12^ samples. In addition, Xiao et al*.*^12^ have compared the three approaches (metagenome, amplicon, and capture). Future guidelines will help in deciding on the best sequencing method to use.

Here, we studied 20 patient nasal swab samples broken down into five series of four samples of equivalent viral load (CT26, CT29, CT32, CT35, and CT36+). In our study, we tested the efficacy of five commercial probe panels for the detection of the SARS-CoV-2 genome, including panels from Illumina, Twist Bioscience, and Arbor Bioscience. Two of the five panels contained only probes specific to the SARS-CoV-2 genome (Twist, Arbor), whereas the other three were pan-virus (Illumina, Twist). These three companies use different carriers for their probes (ssDNA, dsDNA, and ssRNA). Here, we report on the ability of the different kits to detect the SARS-CoV-2 genome, either partially or in its integrity.

## Results

**Figure 1:**
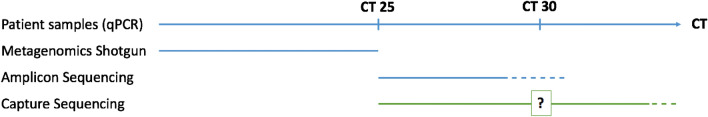
Molecular detection of SARS-CoV-2 by qRT-PCR and SARS-CoV-2 genome sequencing. Blue line: current strategy, green line: potential positioning of the capture technology.

The relative abundance of SARS-CoV-2 genome sequences in patient samples is generally low, requiring overlapping amplicon sequencing or deep shotgun sequencing to accurately detect and reconstruct them. Up until now, the NRC for Respiratory Viruses in France (Institut Pasteur, Paris) has used two approaches to obtain SARS-CoV-2 genomes: shotgun metagenomics for an abundance up to CT25 and amplicon sequencing after CT25. We propose a third and complementary approach using hybridization-capture sequencing (Fig. 1).

### Study design

We performed capture using 20 patient samples positive for SARS-CoV-2 by RT-qPCR to evaluate the efficacy of capture panels to access the SARS-CoV-2 genome in patient samples.

**Figure 2:**
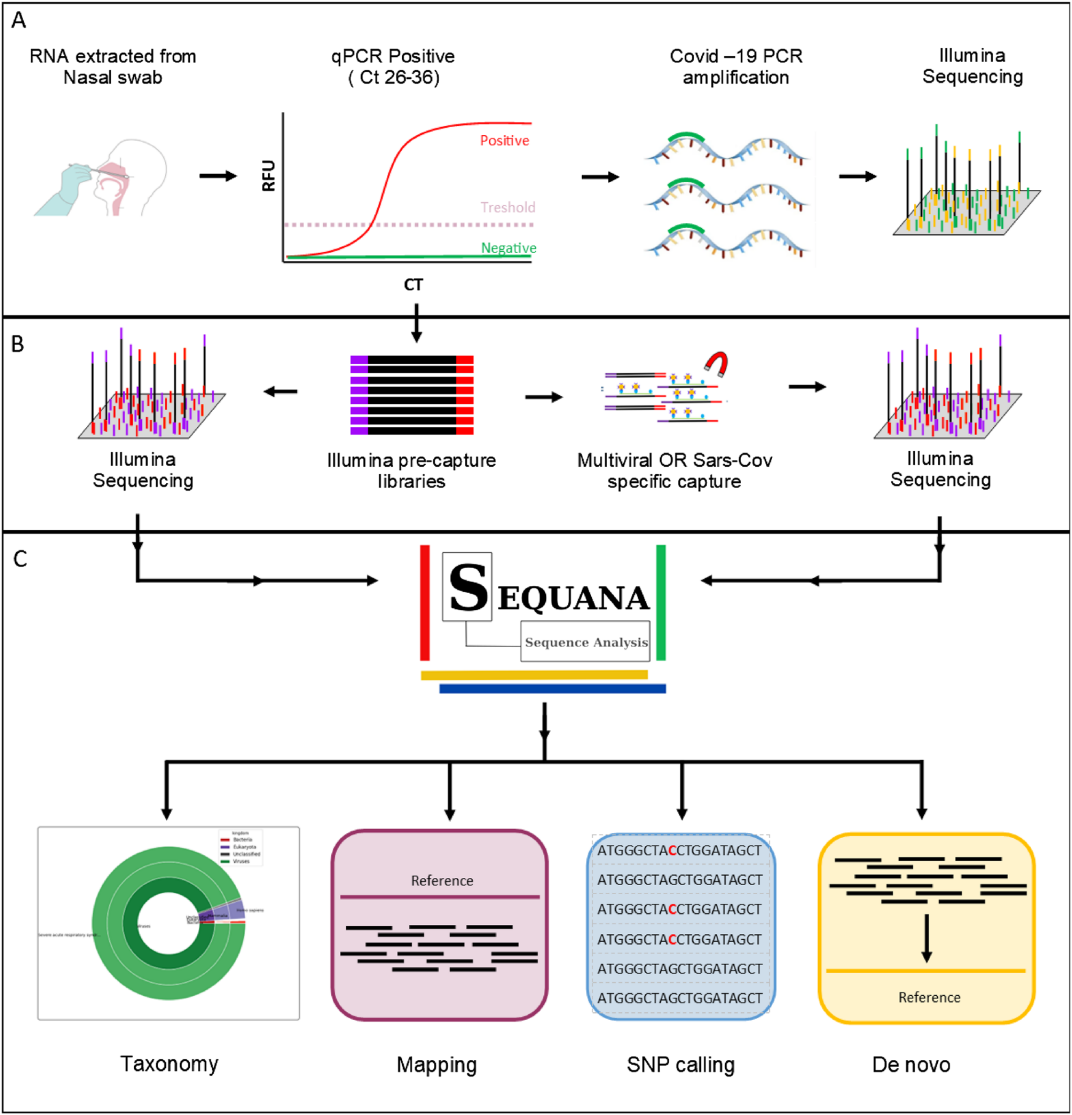
Overview of the study design: (**A**) RNA extraction from 20 samples collected from nasal swabs of patients with symptoms of COVID 19 was performed. After confirmation of the diagnosis by RT-qPCR, the viral genome of SARS-CoV-2 was amplified by PCR. (**B**) From the same RNA extraction, cDNA synthesis was performed using random hexamers. Library preparation was performed using the Nextera Flex Enrichment Library Preparation kit. All pre-capture libraries were sequenced using a Nextseq 500, in high-output mode. Samples were pooled for capture based on their viral CT and captured using five different capture probe pools: three targeting multiple viruses, including the SARS-CoV-2 genome, and two targeting the SARS-CoV-2 viral genome only. All pools were sequenced using a multi-Illumina platform approach. (**C**) All bioinformatics analyses were performed using NGS pipelines available within the Sequana project^13^.

We used five different probe panels: two corresponding to the entire SARS-CoV-2 viral genome and three composed of a mix of different viruses (pan-viral). All panels are ready-to-use commercial designs. For each panel, the capture reactions were performed using the same library preparation for each sample. All libraries were sequenced prior to capture using a NextSeq 500 in high-output mode, paired-end 150 bp. Samples were pooled in groups of four, according to their CT (Table 1), to reduce bias related to target abundance within the samples. We used either a multi-virus panel or a SARS-CoV-2specific panel for the captures. For the multi-virus panels, samples were sequenced using the NextSeq 500 high-output format, paired-end 150 bp, of Illumina. As for the dedicated SARS-CoV-2 panels, all samples were sequenced using a MiSeq V2 paired-end 150 kit (Fig. 2A,B).

**Table 1:** Characteristics of patient nasal-swab samples.

Sample ID	[RNA] ng/µl*	RQN^+^	qRT PCR (CT)	Groups
4885	11	3.2	26.12	A
4716	3.0	4.9	25.77	
4660	1.1	1.4	26.55	
4520	4.0	4.5	25.51	
4707	5.1	4.3	28.57	B
4697	45	1.6	28.72	
4676	0.6	1.3	28.93	
4653	0.8	1.0	29.27	
4861	1.7	1.0	32.95	C
4787	2.5	2.5	32.59	
4688	111	2	31.11	
4673	4.4	1.6	32.6	
4777	2.4	7.7	35.44	D
4668	0.8	2.1	35.88	
4510	Undetectable	Undetectable	34.65	
4489	4.2	1.7	35.15	
4798	0.2	2.5	36.46	E
4797	0.6	3.6	39.19	
4656	0.4	1.5	36.54	
4544	7.8	1.4	36.74	

The 20 patient samples were grouped in 5 categories (A to E) according to their increasing average CTs.

*Nanodrop 1000 and Fragment Analyser.

### Metagenomics analysis of pre-captured samples

We started by sequencing all pre-capture libraries to evaluate the genomic information we could obtain from the samples using this strategy. This is the simplest way to sequence clinical samples, especially when screening for unknown pathogens or co-infections. Although largely used in clinical studies, this method yields high levels of host sequence contamination. Consequently, the genomes of interest may not be detected using such a method.

**Figure 3:**
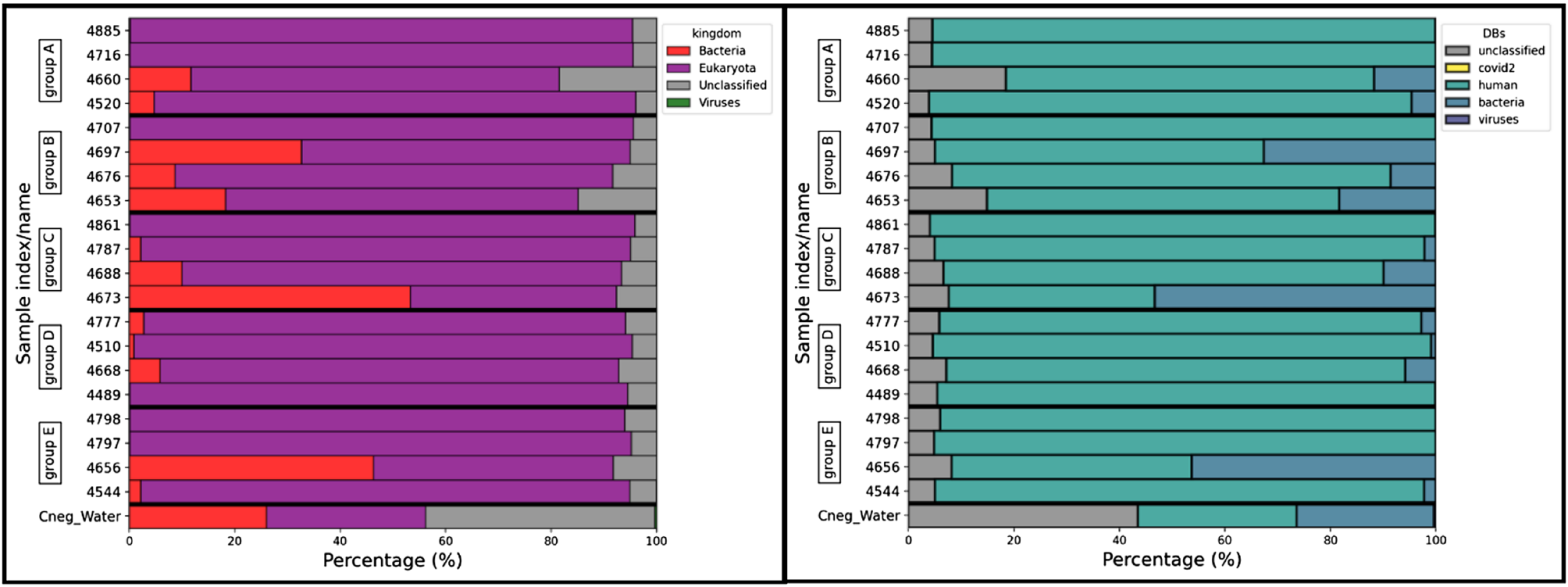
Metagenomic analysis of the pre-capture libraries using Kraken2 software. In the left image, we observe the taxonomic classification at the taxon level. In the right image, several databases were sequentially used, in this order: SARS-CoV-2, human genome, bacteria and viruses. We can observe that most sequences from the 20 samples are classified. Most samples had 95% eukaryotic content (human) and some showed the presence of bacterial sequences of up to 40% (in red on the left panel and blue on the right panel). However, almost no viruses (including SARS-CoV-2) were detected, as represented in both panels.

We obtained an average of 21M reads for each sample (Table 2). Taxonomic analysis detected mostly human sequences, followed by bacteria (Fig. 3, left panel). The presence of viruses was marginal. No reads matched the SARS-CoV-2 genome according to our k-mer analysis (Fig. 3, right panel) confirmed by standard mapping, suggesting that either the samples did not contain any SARS-CoV-2 sequences or that the sequencing depth was insufficient to detect the low-abundance SARS-CoV-2 in our samples.

**Table 2:** Summary of the results of metagenomic sequencing of pre-capture libraries sequenced on the NextSeq 500 in high-output mode.

Sample	Group	Total number of reads	% Reads human	% Reads other	% Reads SARS-CoV-2
4885	A	22.7M	95.3	4.7	0
4716		19.5M	95.5	4.5	0
4660		11.0M	69.9	30	0
4520		16.0M	91.4	8.6	0
4707	B	23.9M	95.5	4.5	0
4697		27.6M	62.4	38	0
4676		16.9M	83.1	17	0
4653		13.9M	66.8	33	0
4861	C	22.3M	95.9	4.1	0
4787		26.9M	92.9	7.1	0
4688		41.0M	83.4	17	0
4673		22.2M	39.1	61	0
4777	D	20.6M	91.4	8.6	0
4668		25.3M	87.0	13	0
4510		9.3M	94.5	5.5	0
4489		19.9M	94.4	5.6	0
4798	E	19.6M	93.9	6.1	0
4797		16.1M	95.1	4.9	0
4656		19.6M	45.5	54	0
4544		25.1M	92.7	7.3	0

Percentages represent the percentage of assignation using k-mer analysis with Kraken2 software. Other: virus + bacteria + unclassified.

Other studies^10–12^ have managed to obtain a full genome from metagenomic sequencing of samples with the same RT-qPCR CTs as those used in this study. However, the number of reads obtained was 70-fold higher than in our study^12^. Although the price per Gb sequenced has dropped significantly, we consider this to not be cost effective and we did not upscale the sequencing strategy of the metagenomic samples.

### Capture results: multi-virus panels

We started by performing capture of all samples with the three multi-virus panels from two different vendors: the Twist Pan-Viral Panel and the Illumina Respiratory Virus Panels. The Twist panel is composed of probes that target 1,160 targets, corresponding to more than 1,000 human pathogenic viruses. Among these viruses, we find the SARS Coronavirus Tor2 (GeneBank ID NC_004718.3), four different Human Coronaviruses (GeneBank IDs NC_002645.1, NC_006577.2, NC_005831.2 and KU131570.1) and Middle East Respiratory Syndrome Coronavirus (GeneBank ID NC_019843.3). At the time of this study, the SARS-CoV-2 genome was not part of this panel. The Illumina panel is composed of 177 targets, corresponding to 83 respiratory viruses and 94 human genes, including the SARS-CoV-2 genome. A list of the accession numbers and gene names is provided in the github repository (https://github.com/biomics-pasteur-fr/manuscript_capture_seq/). As we were performing this benchmark, Illumina released version 2 of its Respiratory virus panel, which showed better overall results than the previous version (Fig. S1A,B). The results described in this paper correspond to those generated using version 2 of the Illumina panel.

#### Twist pan-viral panel

**Figure 4:**
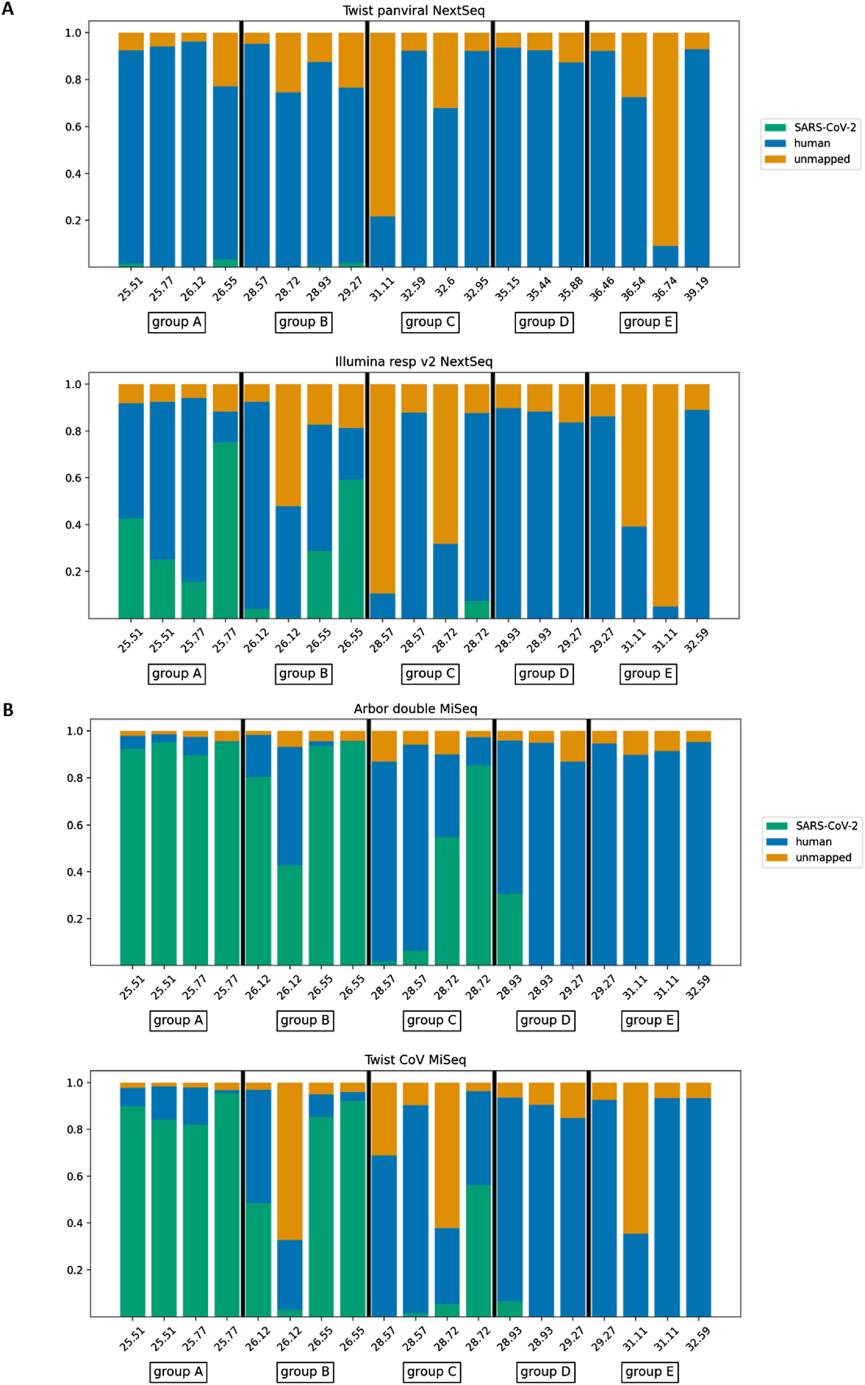
Fraction of reads mapped to SARS-CoV-2 or human genomes for all samples and the four panels tested. (**A**) Fraction of SARS-CoV-2 and human genomes for samples captured using the multi-virus capture panels Twist Pan-viral and Illumina Resv v2. (**B**) Fraction of SARS-CoV-2 and human genomes for samples captured using the SARS-CoV-2 capture panels from Twist and Arbor Biosciences.

The sequencing run of the captured samples using the Twist Pan-Viral Panel generated between 1 and 26M reads per sample (Table 3). As the main goal of this experiment was the detection of SARS-CoV-2 sequences in patient samples, we mapped all data obtained using the SARS-CoV-2 reference genome. For the Twist Pan-Viral Panel, the SARS-CoV-2 virus was poorly captured from samples, with between 0 and 3.2% reads mapped to the reference. The standard metrics used to evaluate the mapping of sequence data in this study are the depth of coverage (DOC hereafter) and breadth of coverage (BOC hereafter)^14^. Even though the DOC for group A ranges between 66 and 506, the BOC is below 40% (Table 3 and Fig. 4A). To further understand the low BOC percentage results we used Sequana Coverage to verify the distribution of the reads along the SARS-CoV-2 genome. We observe in Fig. S4A the coverage of the genome for two samples from group A, 4885 and 4660. We observe that, for both samples, the reads obtained are unequally distributed along the genome. Table 3 shows us that, for both samples, a low BOC is observed, ranging from 25 to 40%. This means that 60–75% of the genome is not covered at all. The parts of the SARS-CoV-2 genome covered by this panel seems to be approximately the same for both samples, even though the depth of coverage for sample 4660 is sevenfold higher than sample 4885. These results suggest a capture bias due to the Coronaviruses genomes used to design this panel. We did a mapping analysis to identify which of the Coronaviruses probes were contributing to the capture of SARS-CoV-2 genome. It appears that the SARS Coronavirus Tor2 probes are the only one able to target SARS-CoV-2 genome (data not shown).

**Table 3:** Mapping results for all samples captured using the different SARS-CoV-2 panels.

Sample	Group	Twist pan-viral panel				Illumina RESV 2 panel				Arbor SARS-CoV panel				Twist SARS-CoV panel			
		Reads (M)	Map (%)	BOC	DOC	Reads (M)	Map (%)	BOC	DOC	Reads (M)	Map (%)	BOC	DOC	Reads (M)	Map (%)	BOC	DOC
4885	A	8.7	0.2	25	66	3.1	16	100	2E + 04	0.4	89	100	2E + 03	0.5	81.7	100	2E + 03
4716		7.4	0.2	29	76	3.1	25	100	3E + 04	0.8	94	100	4E + 03	0.7	83.8	100	2E + 03
4660		3.9	3.3	33	506	4.9	75	100	2E + 05	3.5	94	100	1E + 04	2.6	95.0	100	9E + 03
4520		7.7	1.4	40	441	9.0	43	100	2E + 05	2.5	92	100	1E + 04	2.2	89.6	100	8E + 03
4707	B	9.5	0.0	22	17	4.9	4.1	100	9E + 03	0.8	80	100	3E + 03	0.5	48.4	100	932
4697		5.0	0.0	10	1.4	3.4	0.3	98	516	0.1	43	98	139	0.6	3.0	98	70
4676		5.8	0.4	25	99	3.2	29	100	4E + 04	2.1	92	100	9E + 03	1.4	84.8	100	4E + 03
4653		4.0	1.8	31	281	5.5	59	100	1E + 05	5.8	94	100	2E + 04	5.5	91.6	100	2E + 04
4861	C	4.3	0.1	23	14	1.8	7.5	100	6E + 03	7.7	84	100	3E + 04	3.6	56.1	100	8E + 03
4787		4.9	0.0	4.5	0.2	2.1	0.1	53	51	0.5	6	55	138	1.0	1.4	56	55
4688		19	0.0	1.4	0.1	16	0.0	26	17	0.6	2	27	45	1.7	0.3	28	18
4673		2.6	0.0	6.9	0.9	1.6	0.4	93	249	0.5	54	95	1E + 03	1.5	5.4	94	315
4777	D	7.8	0.0	0.0	0.0	8.5	0.0	0.0	0.0	2.2	0.0	0.0	0.0	2.1	0.0	0.3	0.0
4668		6.7	0.0	0.0	0.0	4.1	0.0	0.0	0.0	1.8	0.0	1.5	0.0	1.3	0.0	1.2	0.0
4510		*–*	*–*	*–*	*–*	*–*	*–*	*–*	*–*	*–*	*–*	*–*	*–*	2.4	46.9	95	4E + 03
4489		7.6	0.0	12	1.2	5.3	0.3	94	776	3.7	30	95	5E + 03	1.7	6.6	94	468
4798	E	2.2	0.0	0.0	0.0	0.5	0.0	0.0	0.0	2.0	0.0	1.2	0.0	1.1	0.0	1.6	0.0
4797		2.6	0.0	0.0	0.0	0.9	0.0	0.0	0.0	3.9	0.0	3.6	0.0	1.4	0.0	0.9	0.0
4656		1.5	0.0	0.0	0.0	0.7	0.0	0.0	0.0	1.1	0.0	0.0	0.0	2.5	0.0	0.0	0.0
4544		26	0.0	0.0	0.0	22	0.0	0.0	0.0	0.4	0.0	0.0	0.0	2.7	0.0	2.7	0.0

All data was mapped to the Wuhan SARS-CoV-2 sequence (accession MN908947.3). The DOC column indicates the depth of coverage (or mean sequencing depth). The BOC column indicates the breadth of coverage (percentage of genome covered by at least one read)^14^.

Interestingly, sample 4489 from group D was enriched for SARS-CoV-2 sequences and had a BOC of 12.2%. This is quite surprising, as all samples with a CT > 32 were not enriched by this panel.

**Figure 5:**
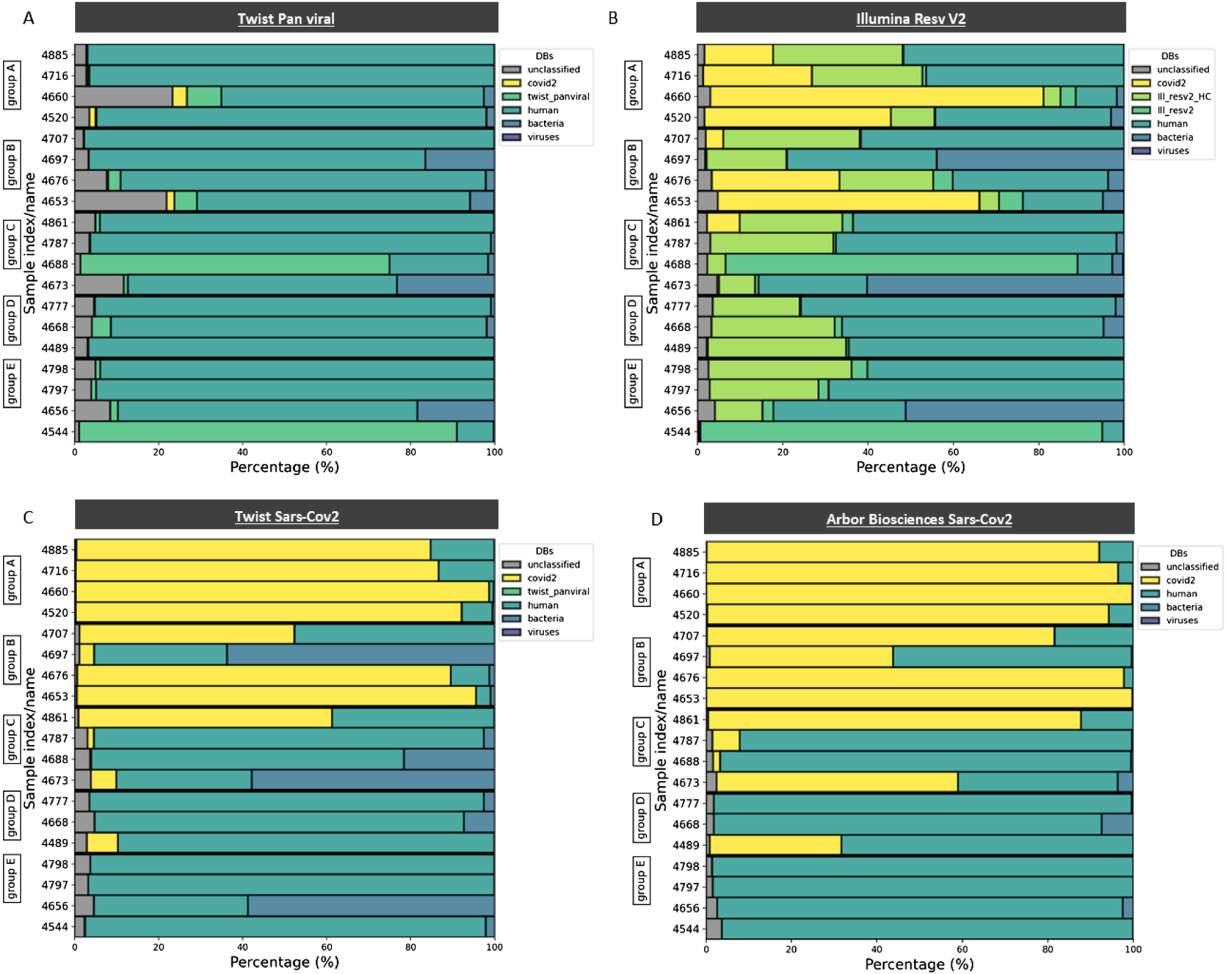
Taxonomic analysis of all panels tested using Kraken2. All reads captured using the four different panels were compared to human, virus, SARS-CoV-2, and bacterial databases. (**A**) For the Twist Pan-Viral capture panel, a supplementary database containing the genome of all viruses from the panel was used. (**B**) For the Illumina Resv V2 panel, two supplementary databases containing the human control genome and the genome of all viruses from the panel were used. (**C**) The Twist SARS-CoV-2 and (**D**) Arbor Biosciences SARS-CoV-2 panels did not require supplementary databases for this analysis. For all panels, all reads not matching any of the databases used were identified as “unclassified”.

We performed taxonomic analysis of all samples to further investigate why we obtained such a small percentage of reads corresponding to our target. The main objective was to classify the reads not mapping to the SARS-CoV-2 reference sequence. With the exception of samples 4544 and 4688, we obtained between 65 and 97% of reads with hits for the human database (Fig. 5A). Interestingly, most of the reads of samples 4544 and 4688 corresponded to *Primate Bocaparvovirus 1* and *Human orthopneumovirus*, respectively. Both viruses are present in the panel and, for sample 4688, we found a number of SARS-CoV-2 reads as well, covering 1% of the entire viral genome. Remarkably, the taxonomic analysis of certain samples also classified a significant proportion of reads into the bacteria kingdom (Fig. 5A). Although they represented only 0.2 to 23% of the reads, they corresponded to different phyla and dozens of different species.

These results suggest that although the viral sequence was captured, it was not captured entirely. Moreover, it is likely that the efficiency of the probes to specifically capture the SARS-CoV-2 reads was low due to the composition of the panel. It is also possible that we lost SARS-CoV-2 information from our samples due to degradation or the library preparation.

#### Illumina respiratory virus panel

The sequencing run of the captured samples using the Illumina Respiratory Virus Panel generated between 3 and 21 M reads per sample. After mapping the reads to the SARS-CoV-2 reference sequence, the samples captured using the Illumina Resv2 Panel showed a higher percentage of reads on target than those captured using the Twist Pan-Viral Panel (Fig. 4A). For samples with a CT < 30 (Groups A/B), the enrichment of SARS-CoV-2 reads was marked (average of 31.4%). For 6 of the 8 samples (in groups A and B), we obtained a BOC of 100% and a DOC of 20,000, meaning that this panel managed to capture the entire viral genome efficiently. Group C showed a lower percentage of enrichment and a lower BOC percentage (between 26 and 99%). DOC have a variable range between 17 and 6000. Groups D and E were not enriched for SARS-CoV-2 sequences, with the exception of sample 4489 (as for the Twist Pan-Viral Panel), which had a BOC of 95% for the SARS-CoV-2 genome with a DOC of 776.

Taxonomic analysis showed between 5 and 69% of the reads mapping to the human database (control genes captured by the panel excluded) (Fig. 5B). Again, most of the reads of samples 4544 and 4688 were identified as *Primate Bocaparvovirus 1* and *Human orthopneumovirus*, respectively. Both viruses are also present in the Illumina panel, confirming the results of the Twist Pan-Viral capture. In addition to *Human Orthopneumovirus*, we detected SARS-CoV-2 reads from sample 4688, covering 26% of the entire viral genome. Using this panel, we also found bacteria in a number of the samples, with the same phyla detected as during the sequencing of the Twist Pan-Viral captured samples.

### SARS-CoV-2 panels

For this benchmark, we tested two different SARS-CoV-2 panels, one from Twist and another from Arbor Biosciences. The main difference between these two panels is that the Arbor probes consist of 2000 ssRNA and those of Twist, 1000 dsDNA. Another difference between those two panels concerns how the capture is performed: Arbor preconizes a double-capture protocol for low-abundance targets, whereas that of Twist is a classic single capture protocol.

#### *Twist* SARS-CoV-2 *capture panel*

Sequencing runs of this capture panel generated between 0.5 and 5M reads per sample. Contrary to the multi-virus kits, between 3 and 94% of the reads mapped to the SARS-CoV-2 reference sequence for samples within groups A and B (Fig. 4B). Consistent with the results obtained with the Illumina multi-viral panel, samples 4707 and 4697 showed a lower percentage of on-target reads than other samples with similar CT values. All samples of these groups had a BOC between 98 and 100% and a DOC ranging from 70 to 20,000. Group C showed between 0.2, 56% on-target reads, and a BOC ranging from 28 to 100% (Table 3). Samples within this group have a lower DOC when compared to groups A and B. With the exception of sample 4489 (as for the multi-virus panels), with 6% on-target reads and a BOC of 94%, samples of groups D and E had few on-target reads, corresponding to 0.3% and 2.7% of the SARS-CoV-2 genome, respectively. Both multi-virus panels tested in this study missed these reads.

Taxonomic analysis of all samples showed between 1 and 96% of the reads classifying as human. As expected, the presence of human reads was higher for samples with high CT values (Fig. 5C). Interestingly, samples 4544 and 4688, for which most of the reads were identified as *Primate Bocaparvovirus 1* and *Human orthopneumovirus*, respectively, with the multi-virus panels, showed no detection of those two viruses after capture with the Twist SARS-CoV-2 Panel. Indeed, 95% of the sample 4544 data matched the human database, and 75% for sample 4688. However, we detected SARS-CoV-2 reads from sample 4688, covering 27% of the entire viral genome, confirming the presence of the virus in this sample. These results suggests high specificity of the Twist SARS-CoV-2 probes to capture the virus. Even though these two samples contained an abundance of other viruses, these probes managed not only to avoid capturing them but also to specifically capture the SARS-CoV-2 reads present in sample 4688. We also observed the presence of bacteria in all samples, between 0.01 and 64%. As shown previously, samples with a high percentage of bacteria did not have a predominant phylum or species, but a mixture of dozens of different species.

#### Arbor SARS-CoV-2 panel

Holmes et al*.*^15^ demonstrated the advantages of double capture when the target genome within samples is scarce. We performed a double capture of our samples using this panel, as recommended by the manufacturer. Globally, this panel showed the best percentage of on-target reads of all panels tested, reaching up to 94% (Fig. 4B). For almost all samples in which the virus was detected, the percentage of on-target reads was higher using the Arbor panel than the Twist panel. Moreover, the BOC results were very similar to those of the Twist SARS-CoV-2 Panel. Samples from groups A, B, and C have a DOC superior than with Twist SARS-CoV-2 Capture panel.

Taxonomic analysis showed between 0.2 and 98% reads matching the human database (Fig. 5D). Although the percentage of reads classified as human was equivalent to that for the Twist SARS-CoV-2 capture panel, there was a significant drop in the percentage of reads classified as bacterial, with a maximum of 7% of reads matching the bacteria database. Concerning samples 4544 and 4688, once again, we did not capture reads corresponding to the other viruses present in these samples. These results show the efficiency of this panel to specifically capture SARS-CoV-2 virus in the presence of other viral genomes. The percentage of reads targeting the human genome was 96% for both samples, suggesting that, in the absence of the target, the probes preferentially bind to sequences of the human genome rather than those of other viral genomes present in the sample.

## Discussion

Here, we assessed the efficiency of several capture panels to capture SARS-CoV-2 viral sequences from patient samples. The panels were chosen based on their specificity (SARS-CoV-2 or pan-viral) as well as their technological difference in the composition of the probes (ssRNA, ssDNA, or dsDNA).

The results obtained by multi-virus capture panels suggest that both panels are able to capture high-abundance targets but fail to capture the lowest-abundance targets, with a high percentage of off-target reads corresponding to the abundance of the host sequences. Illumina Resv2-captured reads correlated to those captured by the SARS-CoV-2-only panels (Fig. S2A–C), in particular from low-CT samples (high viral load). The Illumina Respiratory Virus Panel was modified during this study, mainly to include the SARS-CoV-2 genome on its synthesis. Overall, in terms of SARS-CoV-2 capture from the two multi-virus panels tested, the Illumina Respiratory Virus Panel appears to be the better multi-virus panel for capturing the entire viral genome from patient samples (Fig. S4B). The Twist Pan Viral Panel is useful to do exhaustive screening of samples when co-infections is suspected but not to recover the entire genome of SARS-CoV-2.

Both SARS-CoV-2-only panels were very effective. The Arbor panel showed the highest percentage of on-target reads. However, it should be noted that this panel requires a double-capture protocol. Its effectiveness was especially evident for samples with higher CT values (fewer viral copies). We did not test the Twist SARS-CoV-2 Panel using a double-capture protocol. However, we performed a single capture with the Arbor panel. When a single-capture protocol was used for both panels, the Twist panel actually showed a higher percentage of on-target reads than the Arbor panel for all samples (Fig. S3A–C). Although the percentage of on-target reads suggests a higher capture efficiency, the breadth of coverage was not affected when performing a single or a double capture with the Arbor capture panel (Table S1).

The efficiency of capture of the different panels is affected by different parameters. As observed with the multiviral panels, the genome(s) used to design the probes is crucial to the success of the target capture. Unfortunately, we could not explore further this observation as the probes designs are proprietary information and not available. Indeed, parameters as number of probes and its tilling configuration per region/genome can affect capture efficiency. Twist affirms that its dsDNA probes are more efficient as it captures targets on both strands while other single stranded probes (RNA and DNA) loose half of the information available. When comparing results for both SARS-CoV-2 only panels, we cannot assume the efficiency of Twist SARS-CoV-2 panel is due only to the composition of the probes without considering panel design information.

At the time of this study, there was no variants of concern (VoCs). However, it is known that hybridization reactions should be permissive enough to allow mismatches between the probes and the target. All vendors claim their probes are able to detect viral mutations. Arbor Biosciences probes, for instance, tolerate 20% divergence between probe and target sequence. Even though we could not test the capture panels against VoC contaminated samples, they should perform equally well. Nevertheless, in the case of Omicron VoC, this assertion should be confirmed given the number of mutations, particularly in the Spike region.

Overall, capture followed by sequencing is very effective for the study of SARS-CoV-2 in low-abundance patient samples. Indeed, for samples from groups A and B, we obtained the whole SARS-CoV-2 genome sequence; for groups C (CT 31-33) and D (CT 34-36) a fraction of the genome was obtained when metagenomics analysis failed to even detect the presence of the virus within the samples. Another advantage of the capture method is the low sequencing depth necessary to obtain the whole genome, which reduces not only sequencing costs, but also the ones related to storage, data transfer and computing power necessary for the analysis. Moreover, as discussed above, hybridization capture tolerates both rearrangements and sequence variation, the most recent example being the detection of SARS-CoV-2 sequence using the probes targeting SARS Coronavirus Tor2 from the existing Twist pan viral kit. On the contrary, PCR amplification might need an update of primers to follow the evolution of the virus. In addition, if mutations occur on the regions targeted by the primers, they may be missed during data analysis. As capture panels can be custom synthesized, it is also possible to add specific probes to follow the evolution of the virus if the commercial panels fails to provide satisfactory results. Finally, the multiviral panels were able to detect a co-infection for two of the samples, completely missed by the metagenomics analysis. This information would be also lost if PCR amplification were performed on those samples as primers would be specific for the SARS-CoV-2 genome.

In conclusion, the capture method is suitable to capture SARS-CoV-2 pandemic strains within samples with CT values up to 35, as we observed exploitable signals from the samples of Groups A, B, C, and D.

## Materials & methods

### Patient sample preparation: RNA extraction and qRT-PCR

The samples in this study were nasopharyngeal swabs recovered during the Covid-19 pandemic. They were then pooled and anonymized for viral load testing. RNA extraction and qRT-PCR were carried out by the French National Center for Respiratory Infection Viruses^16^. All methods were carried out in accordance with Covid-19 pandemic guidelines and regulations. All experimental protocols were approved by the French National Center for Respiratory Infection Viruses, Institut Pasteur Paris France. At the time of sampling*, informed consent* *was obtained from all subjects and/or their legal guardian(s). All patients on this study had no* objection to the use of their samples for research purposes.

### Pre-capture library preparation

For all 20 RNA samples, double-stranded cDNA was synthesized using random hexamers and the ProtoScript II first strand cDNA synthesis kit, followed by the NEBNext Ultra II Non-Directional RNA Second Strand Synthesis Module from New England Biolabs. Indexed libraries were prepared using the Nextera Flex for enrichment (Illumina) kit following the manufacturer’s protocol, without modification. The same protocol was followed using RNase-free water (negative control). All pre-capture libraries were quantified using Qubit dsDNA HS kits and qualified using the Agilent Fragment Analyzer HS NGS kit.

### Probe hybridization

Indexed libraries were pooled according to the results of the RT-qPCR CT for SARS-CoV-2 for each sample (Table 1). In total, five capture pools were prepared with four samples in each. Indexed libraries were pooled by mass prior to capture, for a total of 2 µg total DNA per hybridization reaction, respecting the multiplexing strategy described above. For this project, we used five different capture probe panels from three manufacturers: two from Illumina (Respiratory Virus Oligo Panel, ssDNA, 2 version v1 & v2), two from Twist Bioscience (Pan-Viral Panel and SARS-CoV-2 Panel, dsDNA), and one from Arbor Bioscience (SARS-CoV-2 Mybaits Panel, ssRNA). The Illumina hybridization reactions were carried out using the Nextera Flex enrichment protocol as recommended by the manufacturer. For the Twist hybridization reactions, a modified Nextera Flex enrichment protocol was used. Finally, for the Arbor Biosciences hybridization reactions, a double capture was performed according to the probe manufacturer’s protocol.

### Sequencing

The enrichment efficiency for each probe panel was assessed by shotgun sequencing using a NextSeq 500 (Illumina), high-output paired-end 150-bp mode, for all patient libraries prior to hybridization. All captured pools were sequenced using paired-end 150-bp, MiSeq V2 kits. Furthermore, pools captured with the Illumina Respiratory Virus Oligo Panels and Twist Pan-Viral Panel were sequenced on a NextSeq 500 in the high-output, paired-end 150-bp mode. The patient libraries were sequenced on the NextSeq500 sequencers before capture.

### Bioinformatics analysis

All data and sequencing analyses (Fig. 2C) were conducted using dedicated pipelines and scripts available within the Sequana project^13^. Extensive information on how the pipelines were configured can be found as Jupyter notebooks at https://github.com/biomics-pasteur-fr/manuscript_capture_seq.

### Base calling and quality control

All QC and demultiplexing were performed using the dedicated Sequana pipeline^13^ based on FastQC and bcl2fastq software.

### Mapping

Mapping was performed using the sequana_mapper pipeline (version 0.8.5)^13^ with the bowtie2 mapper. In this pipeline, Sequana coverage^17^ was used to help visualize the whole genome and provide statistics. A MultiQC^18^ report also summarizes the multi-sample results. Depending on the question, various reference sequences were used for mapping: (1) SARS-CoV-2 Wuhan-Hu-1 (MN908947_3) to estimate the recovery of SARS-CoV-2 sequences, (2) Illumina and Twist Pan-Viral sequence panels to quantify the efficiency of on-target capture, and (3) human genome Hg38 for quantification and in-silico depletion of human sequences in the samples.

### Taxonomy

We performed a taxonomic classification of all runs using a k-mers approach based on Kraken2^19^. The taxonomic analysis was performed using the Sequana_multitax pipeline^13^, which allows the parallel analysis of all samples using several Kraken databases. The databases were called sequentially using a SARS-CoV-2-only database (DB), followed by a dedicated DB containing the Illumina Respiratory Virus Oligo Panel or the Twist Pan-Viral Panel (“pan” capture only), a DB with the human genome, a DB with bacteria genomes, and, a DB with viruses. This analysis allowed us to classify 95% of the reads, on average. All reports are provided in supplementary data and the HTML reports allow users to examine all runs.We verified that the precision of this approach was high and that the false positive rate remained low. Indeed, we processed 10 different NextSeq and MiSeq runs of samples preceding the Covid-19 crisis using the SARS-CoV-2-only database. No hits were found. Conversely, the addition of SARS-CoV-2 led to a precision of 100%.

### Accession numbers

Sequencing data from the patient samples were depleted of human reads to avoid the dissemination of potentially identifying information. The percentage of each depletion is contained in the additional data. DNA-Seq data generated in this study are available in the Sequence Reads Archives (SRA) with the accession numbers E-MTAB-11232, E-MTAB-11233, E-MTAB-11234, E-MTAB-11235, E-MTAB-11236, and E-MTAB-11237.

### Protocols

Supplementary data contains the protocol used for the capture experiments.

## Supplementary Information

Supplementary Information.
