# Supplementary material for: Capturing SARS-CoV-2 from patient samples with low viral abundance: a comparative analysis
Source: Sci Rep. 2022 Nov 11;12:19274. doi: 10.1038/s41598-022-23422-3 (PMC9652350; doi:10.1038/s41598-022-23422-3)
Supplement: Supplementary file 1 — Supplementary Information. [file 41598_2022_23422_MOESM1_ESM.docx]

*Supplementary Data*

*
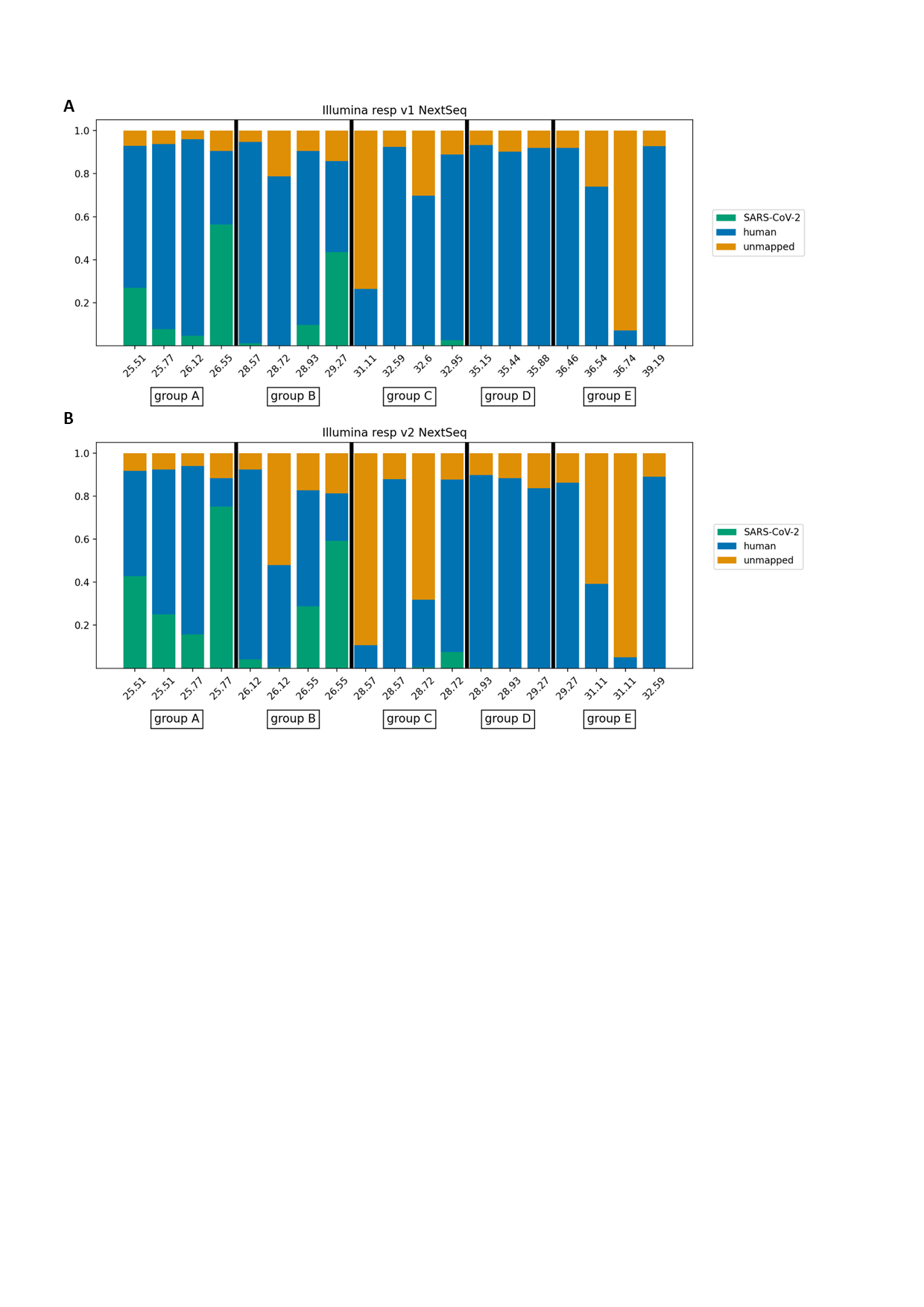
*

*Figure S1. . Comparison between the fraction of on-target reads for Illumina’s Resv 1 panel ( A) and Illumina’s Resv 2 panel (B).*


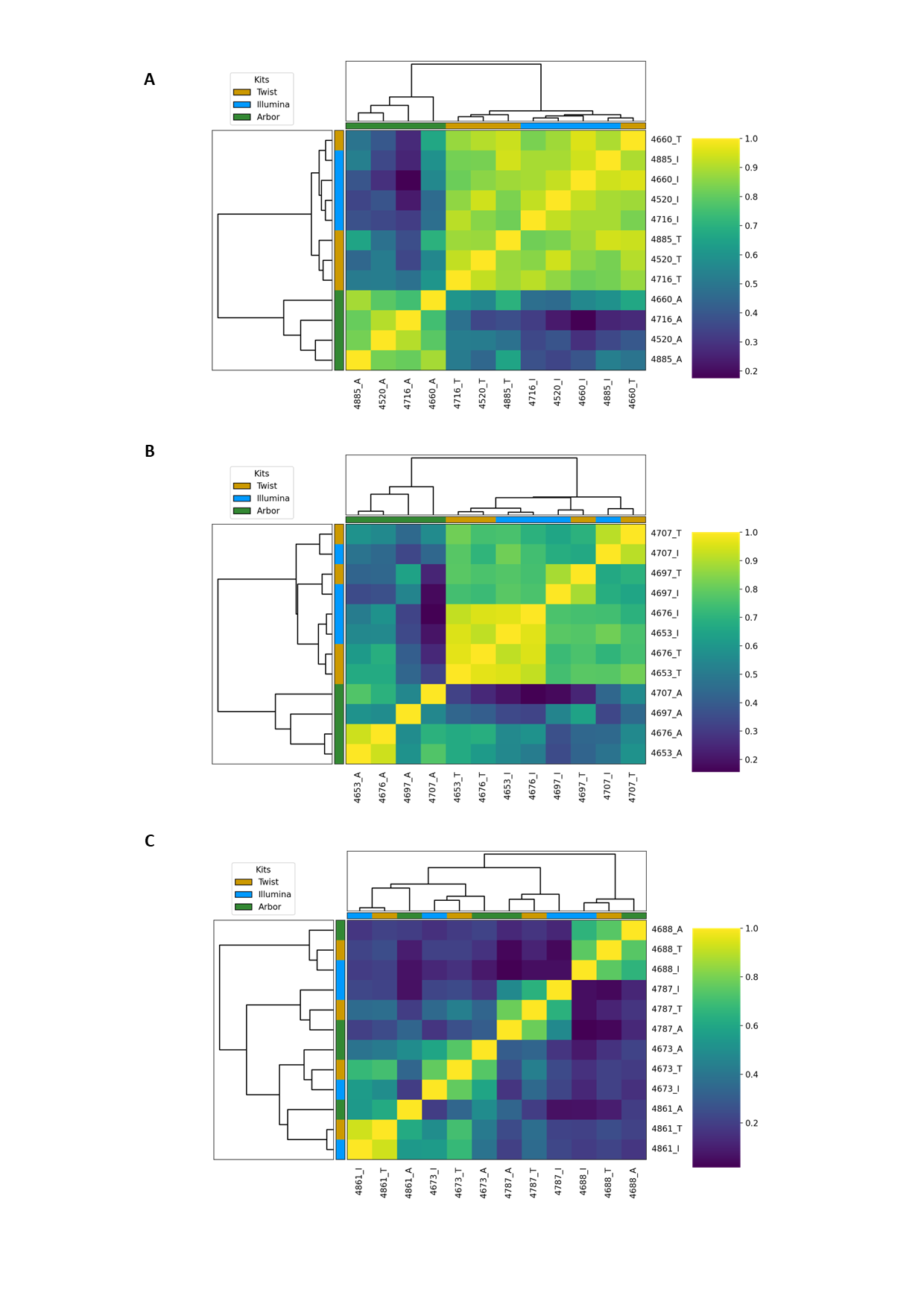


*Figure S2. Correlation between reads from groups A-C samples (A-C), captured with the Twist SARS-CoV-2 in orange, Illumina Resv2 in blue and Arbor Biosciences SARS-CoV-2 in green.*

*
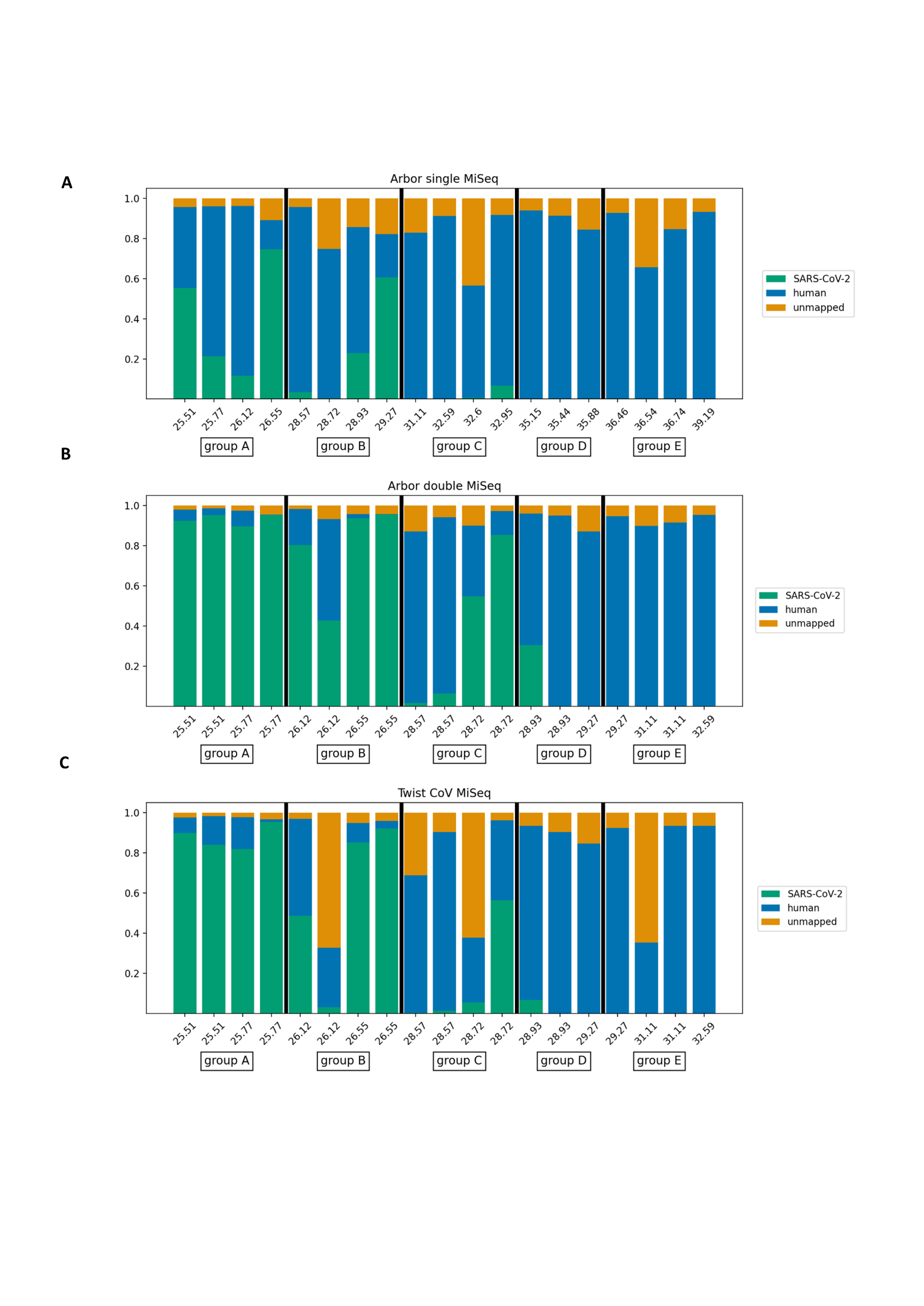
*

*Figure S3. Comparison between the fraction of on-target reads for SARS-CoV-2-only panels in single or double capture. (A) Arbor single capture. (B) Arbor double capture. (C) Twist SARS-CoV-2.*

*
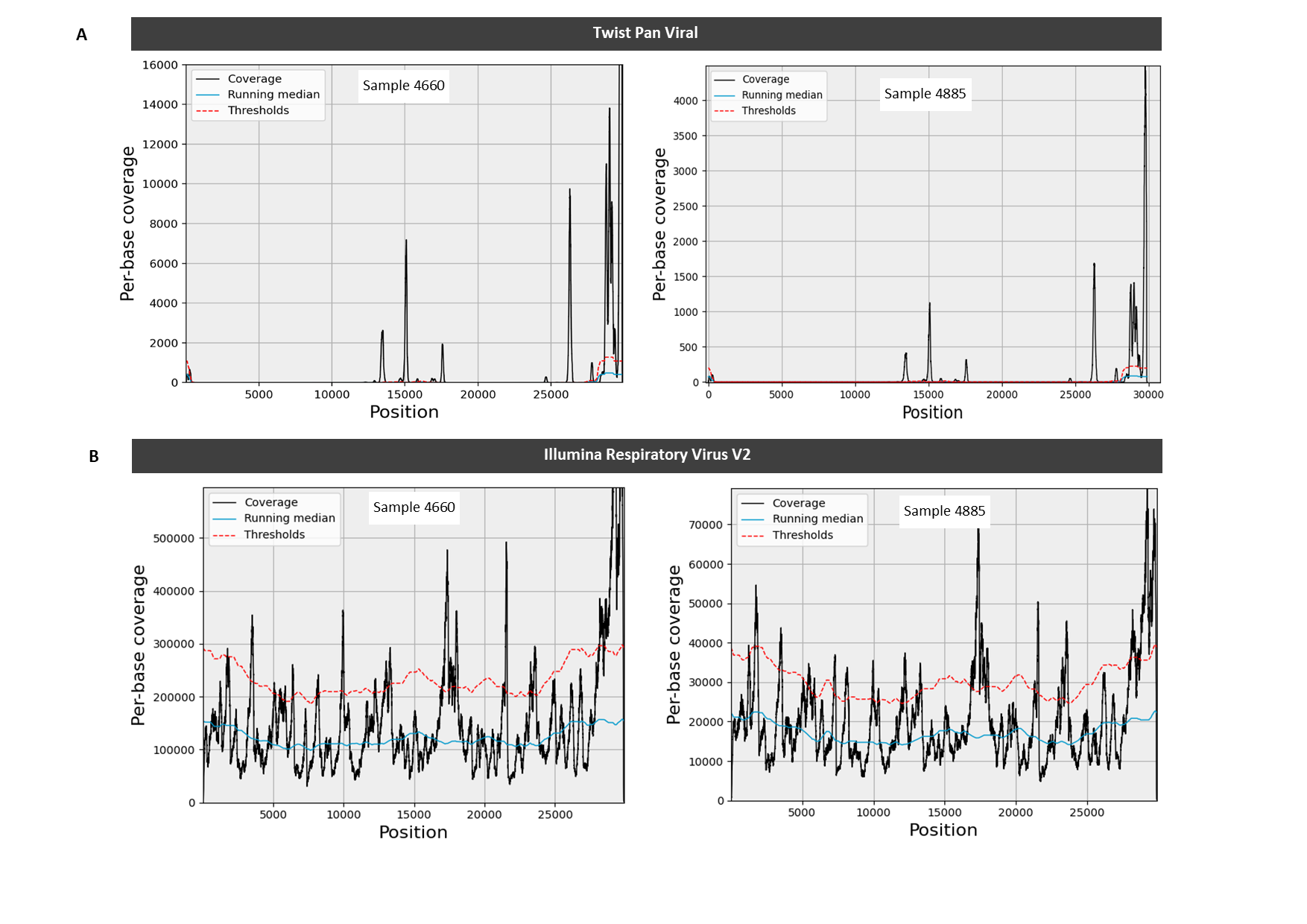
*

*Figure S4. SARS-CoV-2 genome coverage plots for two samples( 4660 and 4485) captured by (A) Twist Pan Viral panel or (B) Illumina Respiratory Virus V2.*

|  |  | Arbor SARS-CoV-2 Panel (1x capture) | | | | Arbor SARS-CoV-2 Panel (2x capture) | | | | Twist SARS CoV-2 Panel | | | |
| --- | --- | --- | --- | --- | --- | --- | --- | --- | --- | --- | --- | --- | --- |
| Sample | Group | Reads (M) | Map (%) | BOC | DOC | Reads (M) | Map (%) | BOC | DOC | Reads (M) | Map (%) | BOC | DOC |
| 4885 | A | 1.5M | 12 | 100 | 742 | 0.4M | 89 | 100 | 2E+03 | 0.5M | 82 | 100 | 2E+03 |
| 4716 |  | 1.6M | 21 | 100 | 2E+03 | 0.8M | 94 | 100 | 4E+03 | 0.7% | 84 | 100 | 2E+03 |
| 4660 |  | 2.0M | 74 | 100 | 6E+03 | 3.5M | 94 | 100 | 1E+04 | 2.6M | 95 | 100 | 9E+03 |
| 4520 |  | 2.2M | 55 | 100 | 5E+03 | 2.5M | 92 | 100 | 1E+04 | 2.2M | 90 | 100 | 8E+03 |
| 4707 | B | 3.0M | 3.4 | 100 | 448 | 0.8M | 80 | 100 | 3E+03 | 0.5M | 48 | 100 | 932 |
| 4697 |  | 1.3M | 0.4 | 97 | 24 | 0.1M | 43 | 98 | 139 | 0.5M | 3.0 | 98 | 70 |
| 4676 |  | 1.8M | 23 | 100 | 2E+03 | 2.1M | 92 | 100 | 9E+03 | 1.4M | 85 | 100 | 4E+03 |
| 4653 |  | 2.3M | 60 | 100 | 6E+03 | 5.8M | 94 | 100 | 2E+04 | 5.5M | 92 | 100 | 2E+04 |
| 4861 | C | 2.7M | 6.6 | 100 | 751 | 7.7M | 84 | 100 | 3E+04 | 3.6M | 56 | 100 | 8E+03 |
| 4787 |  | 2.6M | 0.0 | 50 | 5.4 | 0.5M | 6.3 | 55 | 138 | 1.0M | 1.4 | 56 | 55 |
| 4688 |  | 3.0M | 0.0 | 25 | 1.6 | 0.6M | 1.6 | 27 | 45 | 1.7M | 0.3 | 28 | 18 |
| 4673 |  | 1.1M | 0.6 | 92 | 26 | 0.5M | 54 | 95 | 1E+03 | 1.5M | 5.4 | 94 | 315 |
| 4777 | D | 2.7M | 0.0 | 4.8 | 0.0 | 2.2M | 0.0 | 0.0 | 0.0 | 2.1M | 0.0 | 0.3 | 0.0 |
| 4668 |  | 2.3M | 0.0 | 1.1 | 0.0 | 1.8M | 0.0 | 1.5 | 0.0 | 1.2M | 0.0 | 1.2 | 0.0 |
| 4510 |  | - | - | - | - | - | - | - | - | - | - | - | - |
| 4489 |  | 3.0M | 0.2 | 94 | 32 | 3.7M | 30 | 95 | 5E+03 | 1.7M | 6.6 | 94 | 468 |
| 4798 | E | 2.4M | 0.0 | 2.9 | 0.0 | 1.9M | 0.0 | 1.2 | 0 | 1.1M | 0.0 | 1.6 | 0.0 |
| 4797 |  | 2.9M | 0.0 | 1.7 | 0.0 | 3.9M | 0.0 | 3.6 | 0 | 1.4M | 0.0 | 0.9 | 0.0 |
| 4656 |  | 1.6M | 0.0 | 1.0 | 0.0 | 1.1M | 0.0 | 0.0 | 0 | 2.5M | 0.0 | 0.0 | 0.0 |
| 4544 |  | 0.9M | 0.0 | 7.4 | 0.1 | 0.4M | 0.0 | 0.0 | 0 | 2.7M | 0.0 | 2.7 | 0.0 |

*Table S1. Mapping results for all samples captured by the Arbor SARS-CoV-2 Panel in single and double capture, and the Twist SARS-CoV-2 Panel. All data were mapped to the Wuhan* SARS-CoV-2 sequence *(accession MN908947.3).* *The DOC column indicates the depth of coverage (or mean sequencing depth). The BOC column indicates the breadth of coverage (percentage of genome covered by at least one read) [^14^].*
